# The DNA Methylation Status of Wnt and Tgfβ Signals Is a Key Factor on Functional Regulation of Skeletal Muscle Satellite Cell Development

**DOI:** 10.3389/fgene.2019.00220

**Published:** 2019-03-21

**Authors:** Weiya Zhang, Saixian Zhang, Yueyuan Xu, Yunlong Ma, Dingxiao Zhang, Xinyun Li, Shuhong Zhao

**Affiliations:** ^1^Key Laboratory of Agricultural Animal Genetics, Breeding, and Reproduction of the Ministry of Education, Huazhong Agricultural University, Wuhan, China; ^2^Key Laboratory of Swine Genetics and Breeding of Ministry of Agriculture and Rural Affairs, Huazhong Agricultural University, Wuhan, China; ^3^The Cooperative Innovation Center for Sustainable Pig Production, Wuhan, China

**Keywords:** MeDIP-Seq, DNA methylation, Wnt, Tgfβ, satellite cells, skeletal muscle development

## Abstract

DNA methylation is an important form of epigenetic regulation that can regulate the expression of genes and the development of tissues. Muscle satellite cells play an important role in skeletal muscle development and regeneration. Therefore, the DNA methylation status of genes in satellite cells is important in the regulation of the development of skeletal muscle. This study systematically investigated the changes of genome-wide DNA methylation in satellite cells during skeletal muscle development. According to the MeDIP-Seq data, 52,809–123,317 peaks were obtained for each sample, covering 0.70–1.79% of the genome. The number of reads and peaks was highest in the intron regions followed by the CDS regions. A total of 96,609 DMRs were identified between any two time points. Among them 6198 DMRs were annotated into the gene promoter regions, corresponding to 4726 DMGs. By combining the MeDIP-Seq and RNA-Seq data, a total of 202 overlap genes were obtained between DMGs and DEGs. GO and Pathway analysis revealed that the overlap genes were mainly involved in 128 biological processes and 23 pathways. Among the biological processes, terms related to regulation of cell proliferation and Wnt signaling pathway were significantly different. Gene–gene interaction analysis showed that *Wnt5a*, *Wnt9a*, and *Tgf*β*1* were the key nodes in the network. Furthermore, the expression level of *Wnt5a*, *Wnt9a*, and *Tgf*β*1* genes could be influenced by the methylation status of promoter region during skeletal muscle development. These results indicated that the Wnt and Tgfβ signaling pathways may play an important role in functional regulation of satellite cells, and the DNA methylation status of Wnt and Tgfβ signals is a key regulatory factor during skeletal muscle development. This study provided new insights into the effects of genome-wide methylation on the function of satellite cells.

## Introduction

Skeletal muscle satellite cells are a kind of mononuclear cells with a flattened projection, which locate between the muscle fiber basement membrane and the muscle cell membrane in skeletal muscle (Mauro, 1961). Satellite cells play an important role in skeletal muscle development and regeneration (Ono et al., 2015; Gurevich et al., 2016; Angelino et al., 2018; Guitart et al., 2018). Satellite cells can undergo myogenic differentiation, thereby providing the nucleus for the attached muscle fibers and participating in the development of muscle fibers (Alameddine et al., 1989). In adult skeletal muscle, satellite cells can be activated, and then undergo myogenic differentiation to form new muscle fibers or to fuse with myotubes to repair damaged areas, when muscle fibers are damaged (Collins et al., 2005; Guitart et al., 2018).

Researches showed that the activation, proliferation, and differentiation of satellite cells play a critical role in skeletal muscle development, which require the involvement of a variety of signaling molecules, including TGF pathway (Delaney et al., 2017), FGF pathway (Han et al., 2012), and Wnt pathway (Girardi and Le Grand, 2018). Studies showed that TGFβ1 could inhibit the proliferation of myoblasts and the regeneration of necrotic muscle fibers, and Wnt signaling could regulate the differentiation of myoblast by interacting with TGFβ signaling (Melone et al., 1999; Rudolf et al., 2016). In addition, our previous study showed that TGFβ and WNT9a could inhibit the differentiation of satellite cell, and the synergistic effects of TGFβ2, WNT9a, and FGFR4 signals could be involved in regulating the differentiation of satellite cells during skeletal muscle development (Zhang et al., 2018). However, the epigenetic regulatory mechanism affecting the expression level of these key factors remain largely unknown.

Methylation is a common form of epigenetic regulation affecting the expression of related genes and the development of tissue (Gilsbach et al., 2014). The hypermethylation of promoter regions can lead to gene silencing, but the hypomethylated regions are usually an open area (Bird, 1986; Bernstein et al., 2007). In recent years, many studies have shown that methylation plays an important role in skeletal muscle development, in which satellite cells are involved. Histone methylation and DNA methylation are the two most common forms of methylation modification, and these two forms of methylation modification could effectively affect the function of satellite cells in skeletal muscle development (Liu et al., 2013; Miyata et al., 2017; Robinson and Dilworth, 2018). Methylated *Pax7* could directly bind to MLL1/2 protein and then recruited histone H3K4 methyltransferase complex to regulate the expression of Myf5 (McKinnell et al., 2008; Kawabe et al., 2012). The expression level of *Myogenin* was up-regulated during the differentiation of satellite cells on account of the decrease of the 5′-flanking methylation level (Fuso et al., 2010). Methyl-CpG-binding protein, named CIBZ, could affect the methylation status of the promoter-proximal region of *Myogenin* gene and then inhibited its transcription level (Oikawa et al., 2011). Although many studies have shown that methylation plays an important role in regulating the functions of satellite cells, the systematic exposition of the methylation profile of satellite cells in the process of skeletal muscle development is still very limited.

Our group studied the transcriptome and differentiation characteristics of satellite cells at different developmental stages. We confirmed that the Wnt, Tgfβ, and Fgfr signals were responsible for the attenuation of satellite cell differentiation with development, but the mechanisms of expression change of key factors were unknown (Zhang et al., 2018). Hence, in this study, mouse satellite cells from four time points were obtained. Furthermore, MeDIP-Seq analysis was performed to investigate the genome-wide temporal dynamic changes of DNA methylation during skeletal muscle development. This study systematically analyzed the DNA methylation profile of satellite cells in skeletal muscle development, and identified the potential effects of DNA methylation on skeletal muscle development.

## Results

### Global Mapping of DNA Methylation of Satellite Cells With the Postnatal Development

**FIGURE 1:**
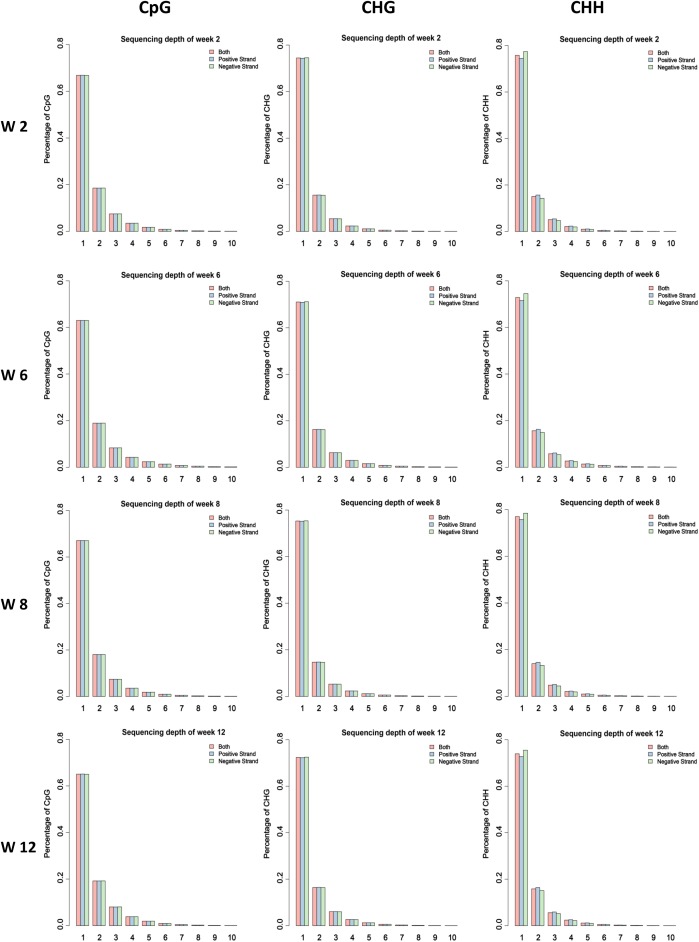
Proportion of the methylated CG, CHG, and CHH sites under different enrichment level of reads in different samples. The average value of each stage was considered.

In our previous study, the skeletal muscle development of mice postnatal can be divided into four stages according to cluster analysis, namely, Weeks 2, 4 & 6, 8, and 10 & 12 (Zhang et al., 2018). Therefore, the leg muscle of four different time points (Weeks 2, 6, 8, and 12) was obtained to isolate satellite cells. Then the MeDIP-Seq analysis was performed. Firstly, we investigated the global DNA methylation status at different developmental stages. According to our data, 21 million clean reads per sample were obtained through filtering the raw reads. Furthermore, the clean reads were mapped to the mouse reference genome, and the mapping rates ranged from 95.74 to 96.34% with uniquely mapped rates ranging from 36.05 to 45.36% (Supplementary Table S1). The uniquely mapped reads were considered during further analysis. The result demonstrating that the global distribution of uniquely mapped reads (chromosomes 1–19 and X) was investigated for each sample (Supplementary Figure S1). Furthermore, the coverage of different methylated models CG, CHG, and CHH were analyzed, demonstrating that the CG, CHG, and CHH sites with high methylation level account for a small proportion in genome-wide (Figure 1 and Supplementary Figures S2–S4).

**FIGURE 2:**
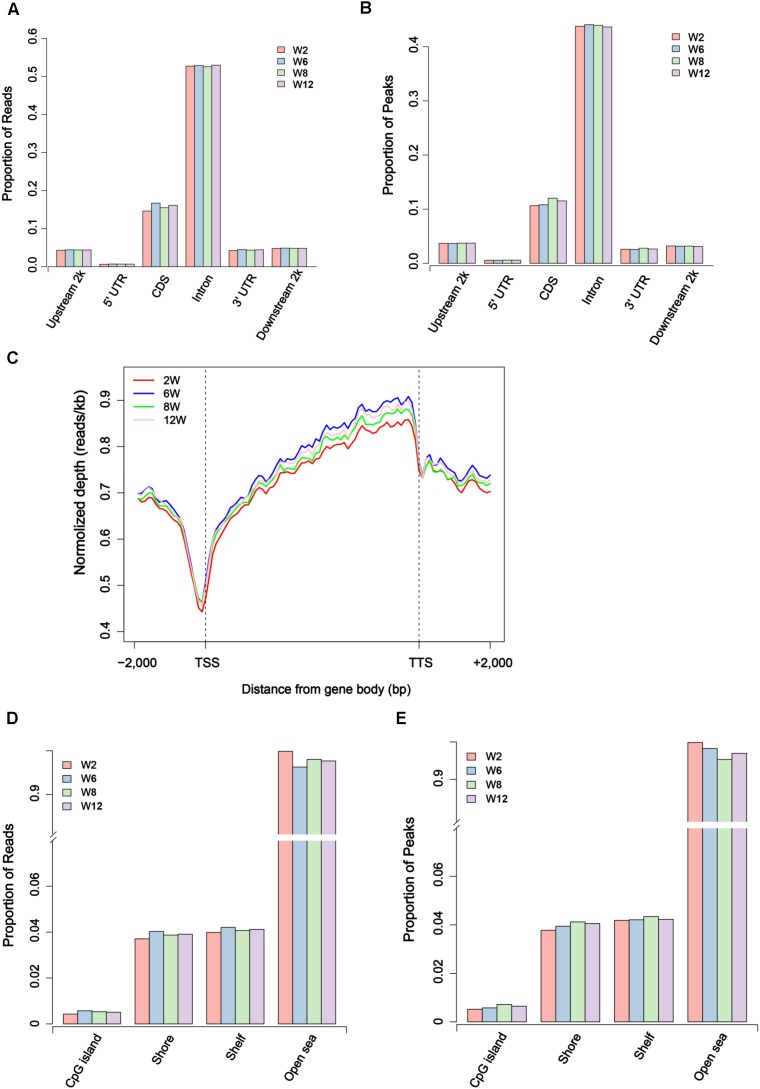
Distribution of unique mapped reads and peaks in different genomic regions. **(A)** The proportion of unique mapped reads in different gene regions. **(B)** The proportion of peaks in different gene regions. **(C)** The distribution of unique mapped reads in 2 kb region upstream of the transcription start site (TSS), the gene body from the TSS to the transcription termination site (TTS), and a 2 kb region downstream of the TTS. **(D)** The proportion of unique mapped reads in CpG island region and outside CpG regions. **(E)** The proportion of peaks in CpG island region and outside CpG regions.

Usually, methylation peak is an important index for the study of differential methylation sites genome-wide. In our study, 52,809–123,317 peaks were obtained for each sample, covering 0.70–1.79% of the genome (Supplementary Table S2). To further investigate the genome-wide distribution of methylation data, the proportion of reads peaks in different genome components was calculated in six different regions. The result showed that the distribution of reads and peaks in CDS regions was second only to intron regions (Figure 2A,B). Furthermore, the specific pattern of gene body was investigated. We divided each gene into 60 equal windows, and the upstream and downstream 2 kb regions were split into 20 non-overlapping windows. The result showed that the reads at the transcription start site (TSS) obviously decreased in all samples but gradually increased in the intragenic region (Figure 2C). Moreover, the proportion of reads and peaks in CpG island was counted. The result showed that the distribution of reads and peaks in CpG island was lower than that outside CpG island regions (Figure 2D,E and Supplementary Figure S5).

### Differential Methylation Regions and Differential Methylation Gene Identification

The methylation data was further explored to compare the differential methylation regions (DMRs) between any two time points (Week 2 versus Week 6, Week 2 versus Week 8, Week 2 versus Week 12, Week 6 versus Week 8, Week 6 versus Week 12, and Week 8 versus Week 12). According to the data, a total of 96,609 DMRs were identified (*p* < 0.01). Among them, 6198 DMRs were annotated into the gene promoter regions (Supplementary Table S3). In this study, we considered the genes with methylation peaks in the promoter regions as the methylated genes. A total of 4726 differential methylation genes (DMGs) were identified (Supplementary Table S4), and then clustering analysis was performed. The result showed that the methylation status of genes in satellite cells was a dynamic process with the development of skeletal muscle (Supplementary Figure S6).

### Functional Clustering Analysis of Overlap Genes Between DMGs and DEGs

**FIGURE 3:**
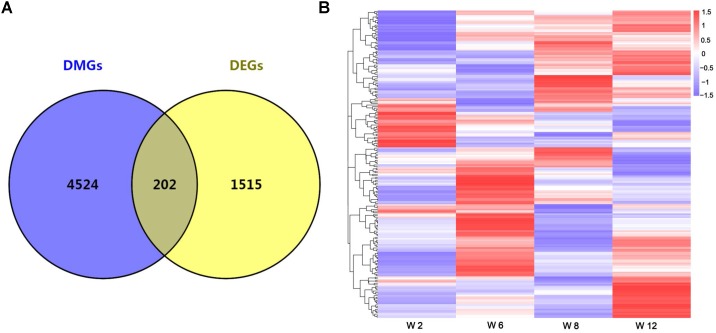
**(A)** The number of overlap genes between DMGs and DEGs is shown by a Venn diagram. **(B)** Cluster analysis of overlap genes between DMGs and DEGs according to the methylation levels of genes. Red indicated hypermethylation levels and purple indicated hypomethylation levels.

Studies showed that the methylation of promoter regions could affect gene expression (Niemann et al., 2008; Laurent et al., 2010). To further understand the effects of methylation on gene function and expression, the differentially expressed genes (DEGs) were compared between any two time points, and the union set of DEGs in any comparison group were considered. A total of 1717 DEGs were identified (FDR < 0.05) (Supplementary Table S4). Furthermore, overlap analysis was performed between DEGs and DMGs, and a total of 202 genes were obtained (Figure 3A and Supplementary Table S4). The result indicated that changes in the expression levels of these genes might be influenced by methylation. Furthermore, the cluster analysis result showed that most genes had a lower methylation level at Week 2, and the methylation level of overlap genes was most significantly different between Weeks 2 and 12 (Figure 3B).

**FIGURE 4:**
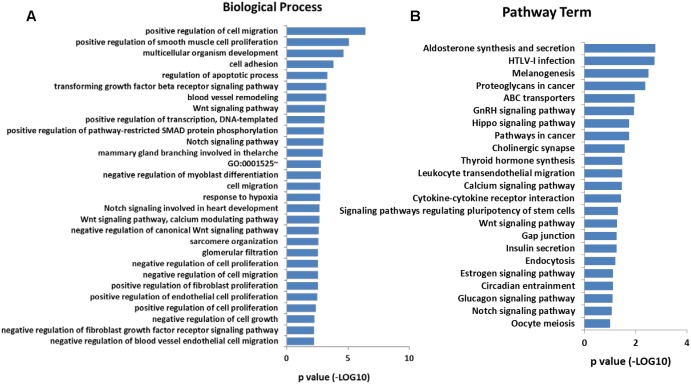
**(A)** Significantly enriched GO terms (top 30) of the overlap genes. **(B)** Significantly enriched signaling pathways of the overlap genes.

In addition, Gene Ontology (GO) analysis was performed using the DAVID program. The result showed that these overlap genes were mainly involved in 128 biological processes (*p* < 0.05). The top 30 GO terms were showed in Figure 4A, including positive regulation of cell migration, positive regulation of smooth muscle cell proliferation, Wnt signaling pathway, calcium modulating pathway, and regulation of cell proliferation. To further understand the role of DMGs in the functional regulation of satellite cells, signaling pathways were analyzed using the Kyoto Encyclopedia of Genes and Genomes database (KEGG). The result showed that the overlap genes were enriched in 23 signaling pathways (*p* < 0.05), some of which were related to muscle development, including Calcium signaling pathway, Wnt signaling pathway, and Insulin secretion (Figure 4B). These results indicated that the DNA methylation status in satellite cells plays an important role in the development of skeletal muscle.

### *Wnt5a*, *Wnt9a*, and *Tgf*β*1* Were the Key Nodes in the Gene Interaction Network

**FIGURE 5:**
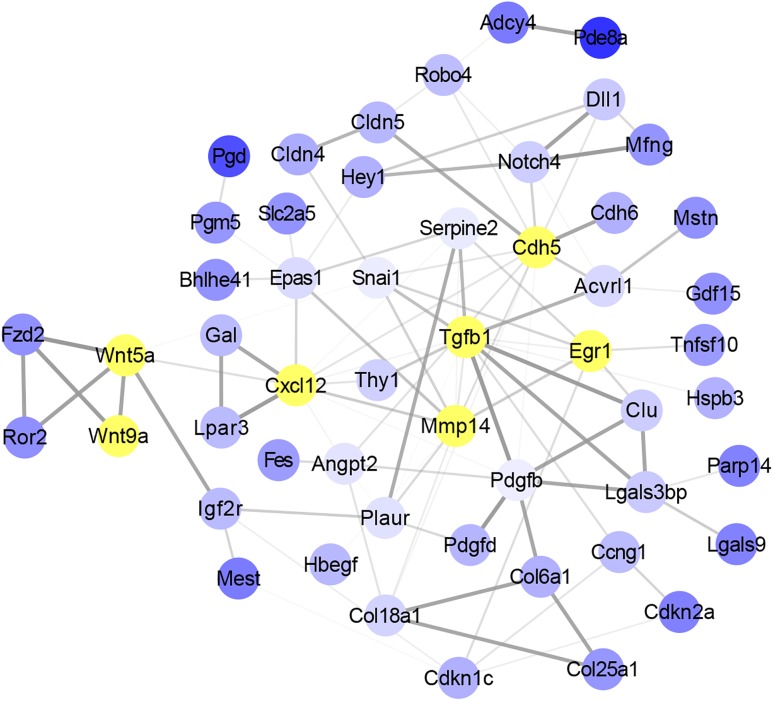
Analysis of the interaction between overlap genes using String software according to interplay index (confidence >0.7). Interplay index between genes was represented by width and transparency of edges. Dark and wide edge indicated high confidence.

To identify the potential key genes that can regulate the function of satellite cells with skeletal muscle development, overlap genes between DMGs and DEGs were selected to study the interaction using String software. A total of 52 genes were considered in the network after filtering genes with weak interaction strength (confidence >0.7) (Figure 5). The network revealed that *Wnt5a*, *Wnt9a*, *Tgf*β*1*, *Cxcl12*, *Mmp14*, *Cdh5*, and *Egr1* were the core nodes. In addition, *Tgf*β*1* had the largest number of interactive genes in the network. Moreover, Wnt signaling pathway, including *Wnt5a*, *Wnt9a*, *Fzd2*, and *Ror2* genes, was located in the upstream of the interaction network and could regulate *Mmp4*, *Cdh5*, and *Egr1* indirectly through the *Cxcl12* gene. This result indicated that *Wnt5a*, *Wnt9a*, and *Tgf*β*1* were the key nodes in the gene interaction network and that the methylation status of Wnt and Tgfβ signaling pathways may play an important role in functional regulation of satellite cells during skeletal muscle development.

### DNA Methylation Could Regulate the Expression of *Wnt5a*, *Wnt9a*, and *Tgf*β*1* Genes at Transcription Level

**FIGURE 6:**
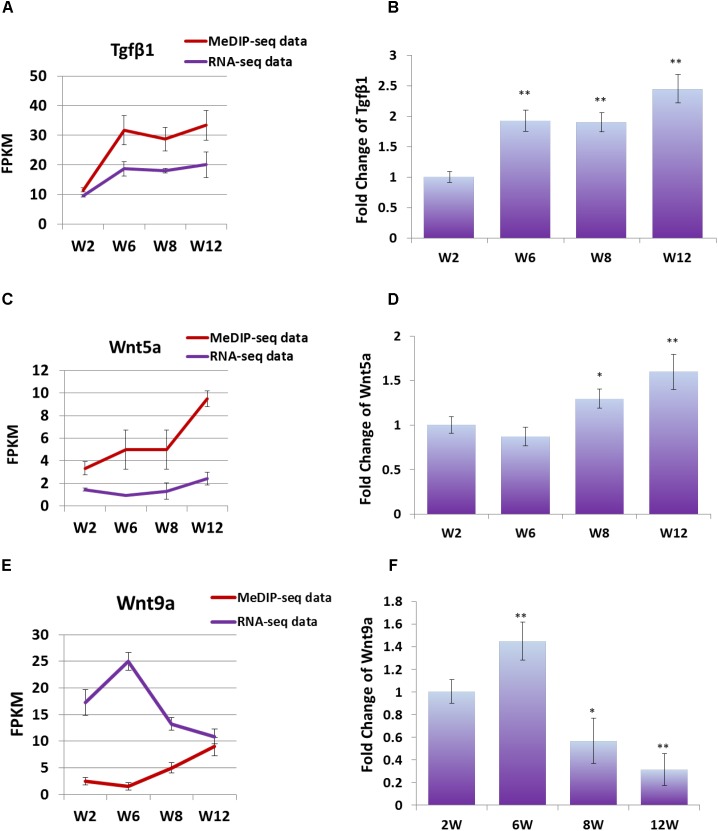
**(A)** FPKM data were used to compare the variation trend of expression level and methylation level of *Tgf*β*1* gene. **(B)** Q-PCR was performed to detect the expression level of *Tgf*β*1* gene in satellite cells. **(C)** FPKM data were used to compare the variation trend of expression level and methylation level of *Wnt5a* gene. **(D)** Q-PCR was performed to detect the expression level of *Wnt5a* gene in satellite cells. **(E)** FPKM data were used to compare the variation trend of expression level and methylation level of *Wnt9a* gene. **(F)** Q-PCR was performed to detect the expression level of *Wnt9a* gene in satellite cells. In Q-PCR experiments, *Tubulin* was used as the internal control, and the relative fold change was compared to the expression in Week 2 satellite cells. Triplicate samples were analyzed for each treatment, and the results were presented as the mean ± SEM. ^∗^*P* < 0.05, ^∗∗^*P* < 0.01.

In order to study the effect of DNA methylation on gene expression level, the trend of gene expression with that of methylation was compared, using FPKM of RNA-seq data and MeDIP-seq data, respectively. The result showed that, the expression trends of *Wnt5a* and *Tgf*β*1* genes were consistent with those of DNA methylation level, which were gradually up-regulated with development (Figure 6A,C). Furthermore, Q-PCR result showed that the expression level of *Wnt5a* and *Tgf*β*1* genes were up-regulated with development (Figure 6B,D). However, the DNA methylation level in the promoter region of *Wnt9a* gene was down-regulated at Week 6, but significantly up-regulated at Weeks 8 and 12, contrary to the change trend of expression level (Figure 6E). Furthermore, Q-PCR result showed that the expression level of Wnt9a gene was significantly up-regulated at Week 6, but down-regulated from Weeks 8 to 12 (Figure 6F). These results indicated that the expression level of *Wnt5a*, *Wnt9a*, and *Tgf*β*1* genes could be influenced by the methylation state of promoter region during skeletal muscle development.

## Discussion

Skeletal muscle satellite cells are a class of stem cells located between the myofibril membrane and the myofibril basement membrane, and they can participate in the development and regeneration of muscle fibers (Charge and Rudnicki, 2004; Lepper et al., 2011). In our previous studies, the capacity of the differentiation of satellite cells decreased with age, and Weeks 4 and 6 were two similar stages of development, as well as Weeks 10 and 12. Moreover, we confirmed that many genes and pathways related to growth and development were involved in this process through transcriptome analysis (Zhang et al., 2018). Therefore, we isolated skeletal muscle satellite cells from mice of different ages for MeDIP-Seq analysis to study the expression change mechanism of related genes during skeletal muscle development. In this study, only four time points were selected to represent the development of skeletal muscle postnatal for the law of skeletal muscle development shown in our previous article. In addition, from Day 1 to Week 2 are the early stages of growth and development after birth. Considering the operability, satellite cells of Week 2 mice were selected as the representative of early stages.

According to the MeDIP-Seq data, 21 million clean reads per sample were obtained by filtering the raw reads. The rate of uniquely mapped ranged from 36.05 to 45.36% (Supplementary Table S1). Studies have shown that methylation peak was an important index of the methylation level (Li et al., 2010; Su et al., 2016). Generally, promoter regions have lower methylation levels than other regions (Laurent et al., 2010; Li et al., 2014). In our study, the genome-wide coverage of peaks was 0.70–1.79%, and the promoter region had a lower methylation level than other regions (Figure 2). Moreover, the proportion of peaks in CpG island regions was lower than that outside CpG island regions due to the small proportion of CpG island regions in genome-wide.

Researches showed that DNA methylation in promoter regions could inhibit the gene expression, namely high methylation levels correspond to low expression levels (Wagner et al., 2014; Wu and Zhang, 2014). In the present study, a total of 96,609 DMRs were annotated into the gene promoter regions, and these corresponded to 4726 DMGs. Moreover, the 202 overlap genes were identified by comparing DMGs and DEGs. Most of the genes were related to satellite cell function and muscle development, including positive regulation of cell migration, positive regulation of smooth muscle cell proliferation, Wnt signaling pathway, calcium modulating pathway, and regulation of cell proliferation. Cluster analysis revealed that many of the overlap genes had a lower methylation levels at Week 2 (Figure 3B). This result indicated that these genes were transcriptionally activated at early stages due to related to the growth and development processes of tissues. Studies showed that dynamics in DNA methylation persisted during development and cell differentiation (Stelzer et al., 2016; Li et al., 2018). Interestingly, the changes of methylation levels of the overlap genes was not linear in these four developmental stages, indicating that these genes may be involved in different biological processes due to the developmental characteristics at the different stages of skeletal muscle development.

Furthermore, signaling pathway analysis was performed. As expected, we found that Wnt signaling pathway was significantly enriched. Researches have shown that Wnt and Tgfβ signaling pathway play important roles in skeletal muscle development regulation (Biressi et al., 2014; Zhu et al., 2017; Girardi and Le Grand, 2018; Zhang et al., 2018). Gene–gene interaction analysis showed that *Wnt5a*, *Wnt9a*, and *Tgf*β*1* were the key nodes in the network. Previous study showed that WNT5a could promote the proliferation of skeletal muscle satellite cells (Otto et al., 2008). Studies showed that TGFβ1 could inhibit the proliferation of myoblasts and the regeneration of myofiber (Melone et al., 1999; Delaney et al., 2017). In addition, our previous study has shown that Wnt and Tgfβ signals could regulate the differentiation of satellite cells. The synergistic effects of TGFβ2, WNT9a, and FGFR4 signals could regulate the differentiation of satellite cells, and WNT9a acted as an intermediate regulator in this equilibrium system (Zhang et al., 2018). Moreover, we also found that *Acvrl1* and *Mstn*, which are members of Tgfβ superfamily (McPherron et al., 1997; Humbert, 2002), also occurred in the interaction network (Figure 5). Studies showed that MSTN could inhibit the differentiation of myoblasts (Guardiola et al., 2012; Rossi et al., 2016). Combining results from our study and those in these researches, we concluded that DNA methylation could affect the function of satellite cells during skeletal muscle development process in mice.

Researches have shown that DNA methylation in promoter regions was related to the silence of gene, while the effect of DNA methylation in gene body regions on gene expression was variable (Gelfman et al., 2013; Wu and Zhang, 2014; Yang et al., 2014). In this study, we focused on the DNA methylation of promoter regions. Study showed that hypermethylation of promoter regions could inhibit the expression of *Wnt9a* gene, which consisted with the result in this study (Figure 6) (Shu et al., 2006). Interestingly, the methylation level in the promoter region of *Wnt5a* and *Tgf*β*1* genes showed positive correlation with their expression level. Therefore, we speculated that the methylation of promoter regions cooperated with that of the gene body regions to regulate the expression of *Wnt5a* and *Tgf*β*1* genes. Hence, we concluded that Wnt and Tgfβ signaling pathways were the key regulators during skeletal muscle development, and their DNA methylation status may be the key factor on functional regulation of satellite cell.

## Conclusion

The DNA methylation in satellite cells dynamically changed during skeletal muscle development, which may affect the expression of genes and regulate the function of satellite cells. The DNA methylation status of Wnt and Tgfβ signals is a key factor on functional regulation of skeletal muscle satellite cell development. This study provides evidence for epigenetic studies of skeletal muscle development. Such information is potentially useful for improving the muscle growth of livestock.

## Materials and Methods

### Mice

All C57BL/6 mice used in this study were obtained from Hubei Center for Disease Control and Prevention (Wuhan, China). All experiments were performed in accordance with the Guide for the Care and Use of Laboratory Animals (Institute of Laboratory Animal Resources, Commission on Life Sciences, National Research Council, 1996). The protocols were approved by the Hubei Province Committee on Laboratory Animal Care (HZAUMU2013-0005).

### Tissue and Cell

Satellite cells were isolated from the hind-limb muscle tissues at four different time points (postnatal Weeks 2, 6, 8, and 12), and 8 ∼ 12 mice (8 mice for Weeks 2 and Week 6, 10 mice for Week 8, and 12 mice for Week 12) were used for each cell isolation experiment. Satellite cell isolation method referred that described in previous study (Lu et al., 2008; Zhang et al., 2018). The muscle tissues were digested for 60–90 min with collagenase I (2 mg/mL) (Sigma, United States, C1639) at 37°C. The dissociated suspension was sifted through 100, 200, and 400 mesh sieves. Then, the suspension was washed with RPMI 1640 medium, re-suspended by growth medium with 15% fetal calf serum (Gibco, United States, 10082-147), chick embryo extract (GEMINI, United States, 100-163p), basic fibroblast growth factor (Life, United States, 13256-029) (0.25 μg/100 mL), and RPMI 1640 medium. The suspension was plated on a normal dish and then transferred to a dish coated with matrigel (BD, United States, 356234) after 2.5 h. The satellite cells were cultured at 37°C in a cell incubator with 5% CO_2_ until they converged to 60%. Then, the second differential attachment experiment was performed.

### RNA-Seq and Data Analysis

The sequencing process and analysis methods used were in accordance with those used in our previous research (Zhang et al., 2018). The total RNA was extracted from isolated satellite cells at six different time points using RNeasy Mini Kit (Qiagen, Germany, 74106) in accordance with the manufacturer’s instructions. Qualified total RNA was further purified by using the RNAClean XP Kit (Beckman Coulter, Inc., Kraemer Boulevard Brea, CA, United States, A63987) and the RNase-Free DNase Set (QIAGEN, Germany, 79254). RNA and the library preparation integrity were verified with an Agilent Bioanalyzer 2100 (Agilent Technologies, Santa Clara, CA, United States). We accomplished the cluster and first dimension sequencing primer hybridization on cBot of Illumina sequencing machine in accordance with the cBot User Guide. Sequencing was performed by Shanghai Biotechnology Corporation (P.R.C). Edger, which is an R package, was used to screen the DEGs.

### MeDIP-Seq

DNA was isolated from cultured satellite cells using OMEGA TISSUE DNA Kit (2000) (*n* = 3 for each time point). The concentration and quality of DNA were assessed by NanoDrop^®^ ND-1000 and agarose gel electrophoresis. The library was prepared according to MeDIP library development flow. Sequencing was performed by SHANGHAI BIOTECHNOLOGY CORPORATION. The amount of DNA used for sequencing was about 2 μg. The sequencing process included the following: (1) Library construction was performed and the integrity was verified with Agilent 2100 (Agilent Technologies, Santa Clara, CA, United States). (2) Cluster generation and primer hybridization were completed on the cBot equipped with the Illumina HiSeq sequencing instrument according to the corresponding process shown in the cBot User Guide. (3) The sequencing process was controlled by data collection software provided by Illumina, and real-time data analysis was carried out.

### Analysis of MeDIP-Seq Data

Raw sequencing data were first filtered with the following five steps: (a) Reads with >50% bases having phred quality < 20 were removed; (b) Removal of the bases with phred quality < 20 in 3′ end; (c) Removal of adapter contained in reads; (d) Removal of reads with length < 20; (e) Removal of reads that contain ‘N’ base.

Clean reads were aligned to the Ensembl mouse reference genome (GRCm38^1^) using BWA (Version 0.7.17) with default parameters (Li and Durbin, 2009). To investigate the general model of methylation, we analyzed the distribution of aligned reads on the whole reference genome, CGIs, and gene regions, as well as the genome coverage of CG, CHG, and CHH regions with different sequencing depths in each sample and group. The peak distribution was analyzed using MACS (version 2.1.1) software in different genome components, including upstream 2 kb, 5′ UTR, CDS, intron, 3′ UTR, downstream 2 kb, and CpG islands (Zhang et al., 2008).

### DMRs and DMGs Identify

EdgeR integrated in the R packages MEDIPS with 200 bp window size was used to identify DMRs between different groups (Lienhard et al., 2014). MEDIPS identified DMRs between groups by calculating Wilcoxon rank tests for the reads per million (rpm) values of each window (cite MEDIPS). DMRs were filtered for windows with *p* < 0.01. Further, ChIPseeker was performed to annotate the DMRs in gene level, and only the genes with promoter annotated by DMRs were defined as differential methylated genes (DMGs) (Yu et al., 2015). All DMGs were subsequently analyzed with GO and KEGG pathway of the DAVID web server (Huang da et al., 2009).

### Q-PCR

Reverse transcription was performed to initiate cDNA synthesis by using the Prime Script^TM^ RT Reagent Kit with gDNA Eraser (TAKARA BIO INC, Otsu, Shiga, Japan). THUNDERBIRD SYBR qPCR Mix (TOYOBO, Japan) was used for Q-PCR, and the results were monitored using a CFX384 Real-Time PCR Detection System (Bio-Rad, United States). The sequences for Q-PCR primer were as follow: Wnt9a-F, 5′GATTTGCGAGCCCGAGTG3′, Wnt9a-R, 5′GTCTCATATTTGTGTTTTAGGTGCTT3′; Wnt5a-F, 5′CTCACCCCCACAGGCAAC3′, Wnt5a-R, 5′TGCCCTACCAGCAGTGAGTG3′; Tgfβ1-F, 5′GGCGGTGCTCGCTTTGTA3′, Tgfβ1-R, 5′TCCCGAATGTCTGACGTATTGA3′.

## Data Availability

The data sets supporting the results of this article were included within the article and additional files. Raw sequencing reads are available at the Short Read Archive (SRA) database of NCBI (bio-project accession PRJNA510174, study SRP173522, MeDIP-seq accessions SRR8325050-SRR8325061, and RNA-seq accessions SRR8453587-SRR8453598).

## Author Contributions

WZ conducted the experiments and prepared the materials involved in this study. SaZ and YX performed the bioinformatics analysis. ShZ and XL conceived this study. ShZ, XL, and WZ participated in its design and coordination. XL, WZ, and SaZ contributed to the analysis and interpretation of the data. WZ drafted the manuscript. ShZ, XL, YM, and DZ helped to revise the manuscript. All authors read and approved the final manuscript.

## Conflict of Interest Statement

The authors declare that the research was conducted in the absence of any commercial or financial relationships that could be construed as a potential conflict of interest.

## Footnotes

1 ftp://ftp.ensembl.org/pub/release-94/fasta/mus_musculus/dna/
