# Supplementary material for: The DNA Methylation Status of Wnt and Tgfβ Signals Is a Key Factor on Functional Regulation of Skeletal Muscle Satellite Cell Development
Source: Front Genet. 2019 Mar 21;10:220. doi: 10.3389/fgene.2019.00220 (PMC6437077; doi:10.3389/fgene.2019.00220)
Supplement: Supplementary file 1 [file Table_1.DOCX]

**Table S1. Overview of the data of MeDIP-seq.**

| Sample | | Data (bp) | | Total number of reads | Q30 ratio (%) | Q20 ratio (%) | GC content (%) | Total mapped reads | Percentage of mapped reads (%) | Total unique mapped reads |
| --- | --- | --- | --- | --- | --- | --- | --- | --- | --- | --- |
| W 2 | W 2-1 | | 1209997905 | 24258831 | 0.96 | 0.98 | 0.40 | 23225046 | 0.96 | 8745892 |
|  | W 2-2 | | 1079780005 | 21652728 | 0.95 | 0.98 | 0.40 | 20736367 | 0.96 | 7959075 |
|  | W 2-3 | | 1071924614 | 21500180 | 0.96 | 0.98 | 0.42 | 20691202 | 0.96 | 9536076 |
| W 6 | W 6-1 | | 1100638593 | 22076748 | 0.96 | 0.98 | 0.42 | 21236398 | 0.96 | 9484608 |
|  | W 6-2 | | 916525684 | 18386384 | 0.95 | 0.98 | 0.42 | 17713595 | 0.96 | 8340893 |
|  | W 6-3 | | 1138744066 | 22842203 | 0.95 | 0.98 | 0.42 | 21963530 | 0.96 | 9931402 |
| W 8 | W 8-1 | | 901590425 | 18080314 | 0.96 | 0.98 | 0.41 | 17389814 | 0.96 | 7603206 |
|  | W 8-2 | | 1089881199 | 21862688 | 0.95 | 0.98 | 0.42 | 21042094 | 0.96 | 9528655 |
|  | W 8-3 | | 1058859466 | 21238903 | 0.95 | 0.98 | 0.41 | 20383640 | 0.96 | 8558036 |
| W 12 | W 12-1 | | 1040772202 | 20874128 | 0.95 | 0.98 | 0.42 | 20078756 | 0.96 | 8637723 |
|  | W 12-2 | | 1029474088 | 20648874 | 0.95 | 0.98 | 0.42 | 19845980 | 0.96 | 8629762 |
|  | W 12-3 | | 1074779439 | 21552479 | 0.96 | 0.98 | 0.41 | 20695290 | 0.96 | 8580007 |
