# Supplementary material for: The DNA Methylation Status of Wnt and Tgfβ Signals Is a Key Factor on Functional Regulation of Skeletal Muscle Satellite Cell Development
Source: Front Genet. 2019 Mar 21;10:220. doi: 10.3389/fgene.2019.00220 (PMC6437077; doi:10.3389/fgene.2019.00220)
Supplement: Supplementary file 2 [file Table_2.DOCX]

**Table S2. The information of peaks of MeDIP-seq data.**

| Sample | | Total number of peaks | Peak mean length | Peak total length | Peak coverd size in genome（%） |
| --- | --- | --- | --- | --- | --- |
| 2W | 2W-3-1 | 87158 | 323.76 | 28218510 | 1.51 |
|  | 2W-3-3 | 72523 | 325.86 | 23632272 | 1.26 |
|  | 2W-4-1 | 96980 | 268.76 | 26064695 | 1.39 |
| 6W | 6W-1 | 117092 | 269.62 | 31570315 | 1.69 |
|  | 6W-2 | 89457 | 268.03 | 23976724 | 1.28 |
|  | 6W-3 | 123317 | 271.85 | 33523957 | 1.79 |
| 8W | 8W-2 | 52809 | 246.15 | 12999135 | 0.70 |
|  | 8W-7 | 114674 | 270.40 | 31007502 | 1.66 |
|  | 8W-8 | 77056 | 326.10 | 25127942 | 1.34 |
| 12W | 12W-6 | 90423 | 266.34 | 24083197 | 1.29 |
|  | 12W-7 | 92122 | 272.80 | 25130423 | 1.34 |
|  | 12W-8 | 77787 | 260.96 | 20299531 | 1.09 |
