# Supplementary material for: The DNA Methylation Status of Wnt and Tgfβ Signals Is a Key Factor on Functional Regulation of Skeletal Muscle Satellite Cell Development
Source: Front Genet. 2019 Mar 21;10:220. doi: 10.3389/fgene.2019.00220 (PMC6437077; doi:10.3389/fgene.2019.00220)
Supplement: Supplementary file 3 [file Table_3.DOCX]

**Table S3. The number of DMRs in different groups.**

| Samples | DMR number | | |
| --- | --- | --- | --- |
|  | Up | Down | Total |
| W 2 vs W 6 | 412 | 760 | 1172 |
| W 2 vs W 8 | 545 | 403 | 948 |
| W 2 vs W 12 | 583 | 673 | 1256 |
| W 6 vs W 8 | 572 | 323 | 895 |
| W 6 vs W 12 | 596 | 401 | 997 |
| W 8 vs W 12 | 444 | 486 | 930 |
| Total | 3152 | 3046 | 6198 |
