# Supplementary material for: The DNA Methylation Status of Wnt and Tgfβ Signals Is a Key Factor on Functional Regulation of Skeletal Muscle Satellite Cell Development
Source: Front Genet. 2019 Mar 21;10:220. doi: 10.3389/fgene.2019.00220 (PMC6437077; doi:10.3389/fgene.2019.00220)
Supplement: Supplementary file 5 [file Data_Sheet_1.docx]

**Supplemental figure legends**

Figure S1. Chromosome distribution of reads in different samples.

Figure S2. Proportion of the methylated CG sites under different enrichment level of reads in different samples.

Figure S3. Proportion of the methylated CHG sites under different enrichment level of reads in different samples.

Figure S4. Proportion of the methylated CHH sites under different enrichment level of reads in different samples.

Figure S5. (A) The number of unique mapped reads in different gene regions. (B) The number of peaks in different gene regions.

Figure S6. Cluster analysis of all DMGs in the four time points.
