# Supplementary figures and images for: The DNA Methylation Status of Wnt and Tgfβ Signals Is a Key Factor on Functional Regulation of Skeletal Muscle Satellite Cell Development
Source: Front Genet. 2019 Mar 21;10:220. doi: 10.3389/fgene.2019.00220 (PMC6437077; doi:10.3389/fgene.2019.00220)

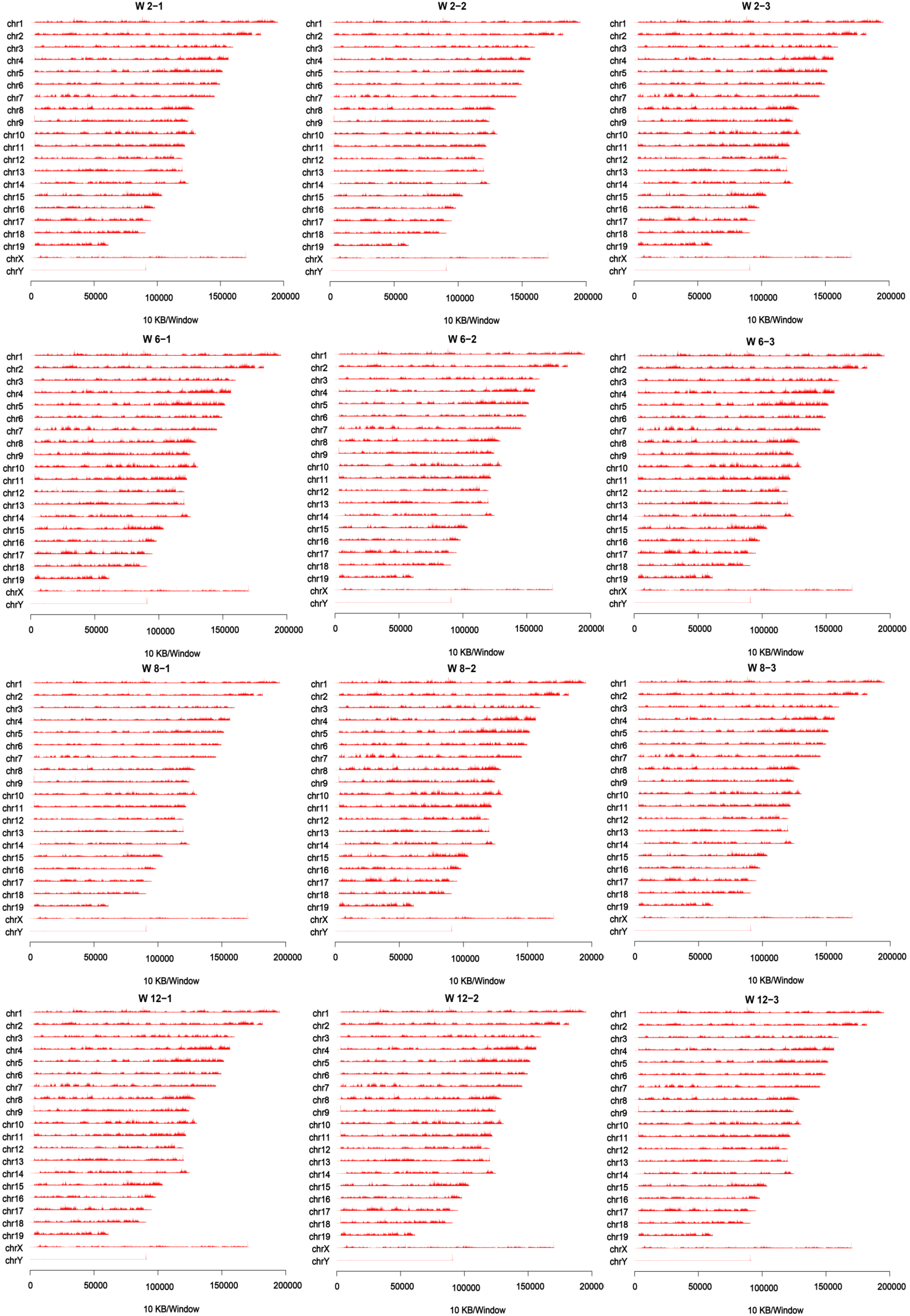

Supplement: Supplementary file 6 [file Image_1.TIF]

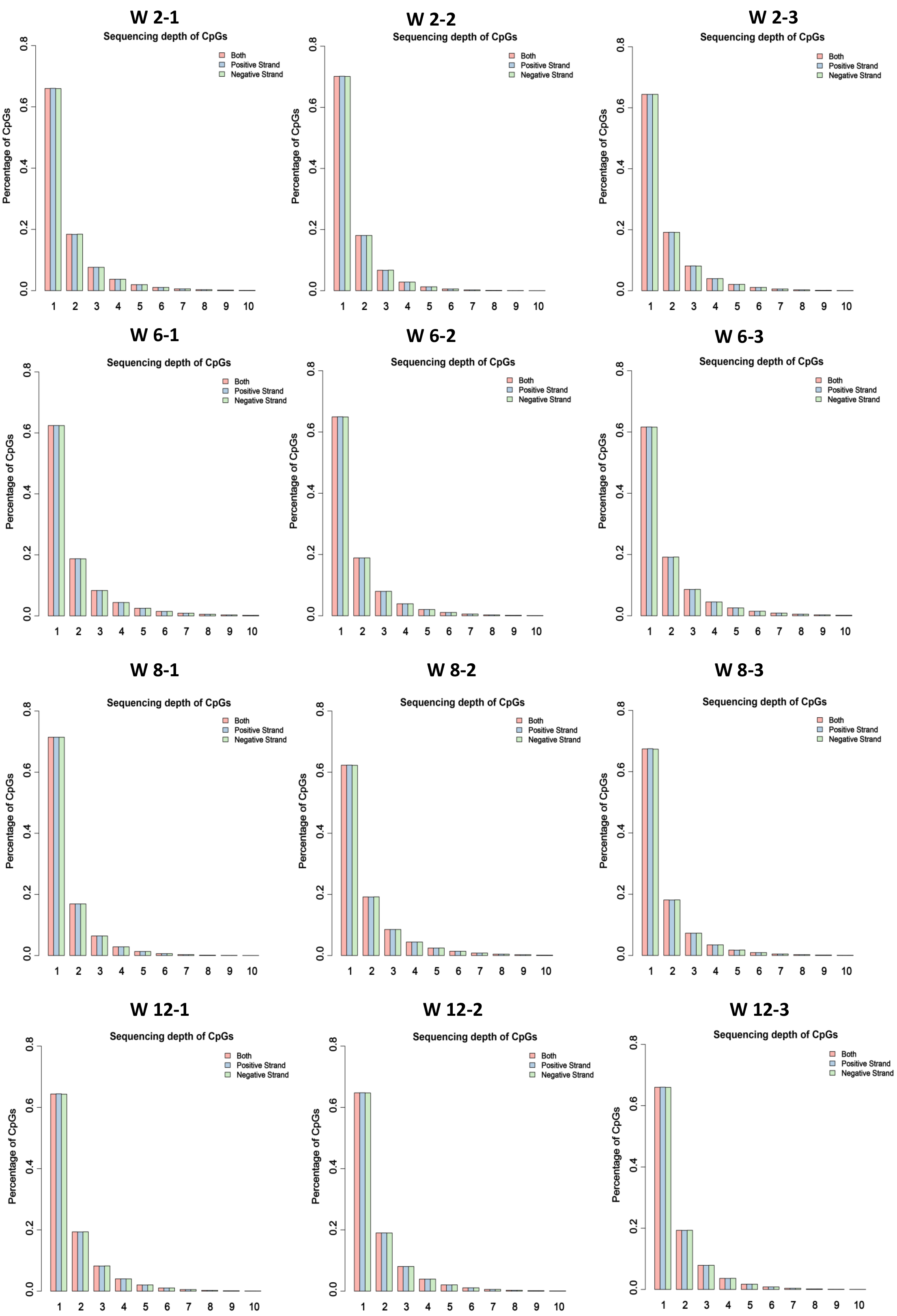

Supplement: Supplementary file 7 [file Image_2.TIF]

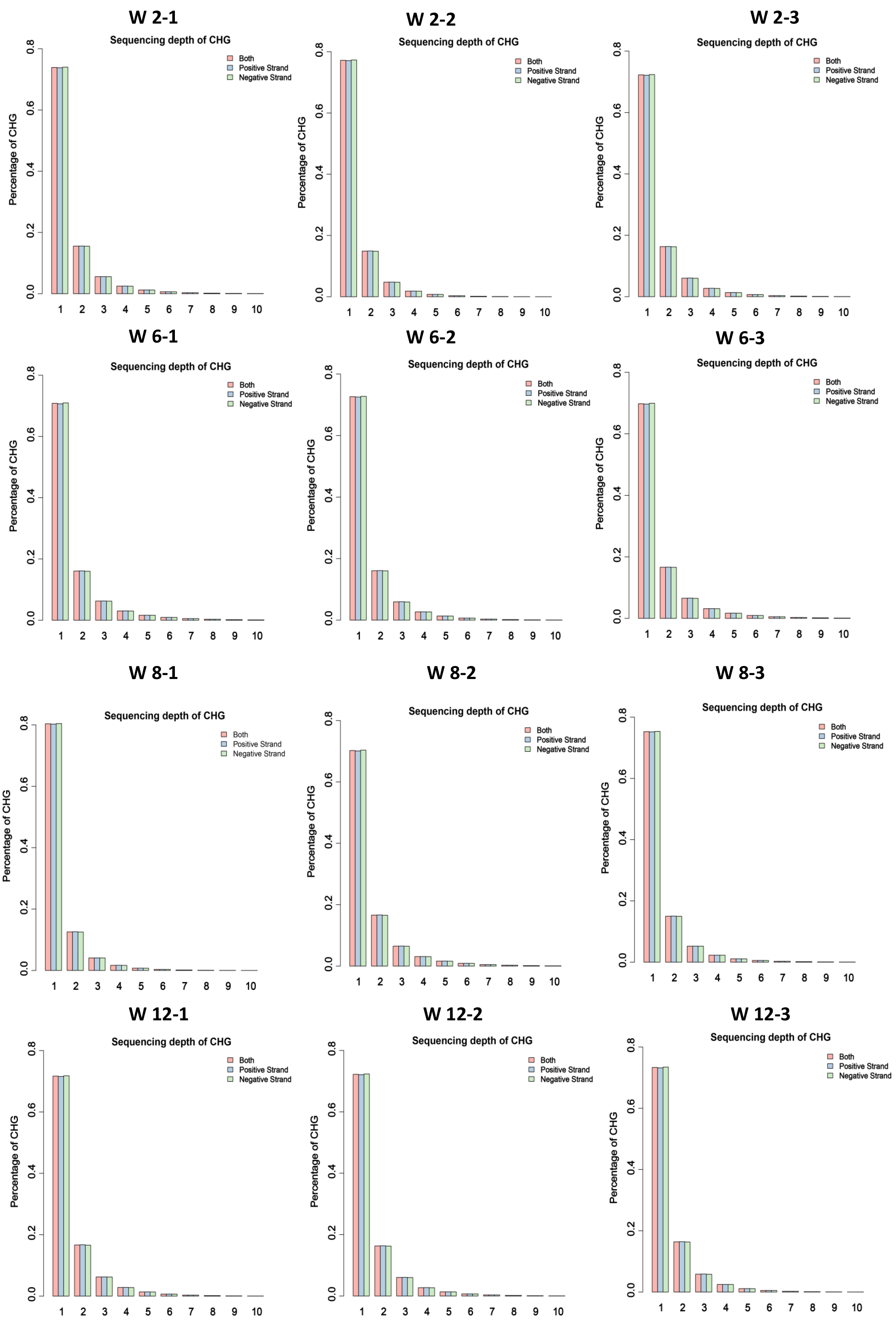

Supplement: Supplementary file 8 [file Image_3.TIF]

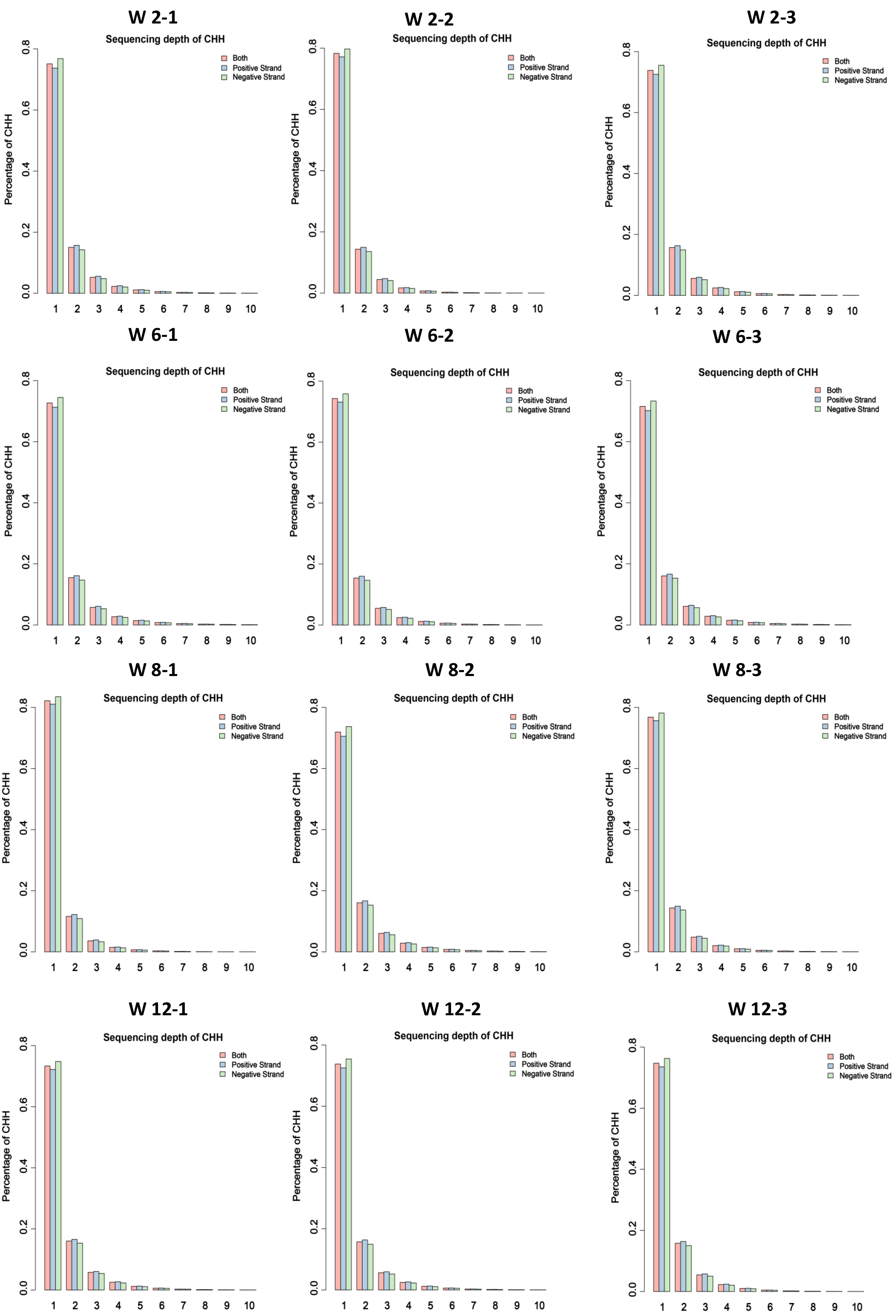

Supplement: Supplementary file 9 [file Image_4.TIF]

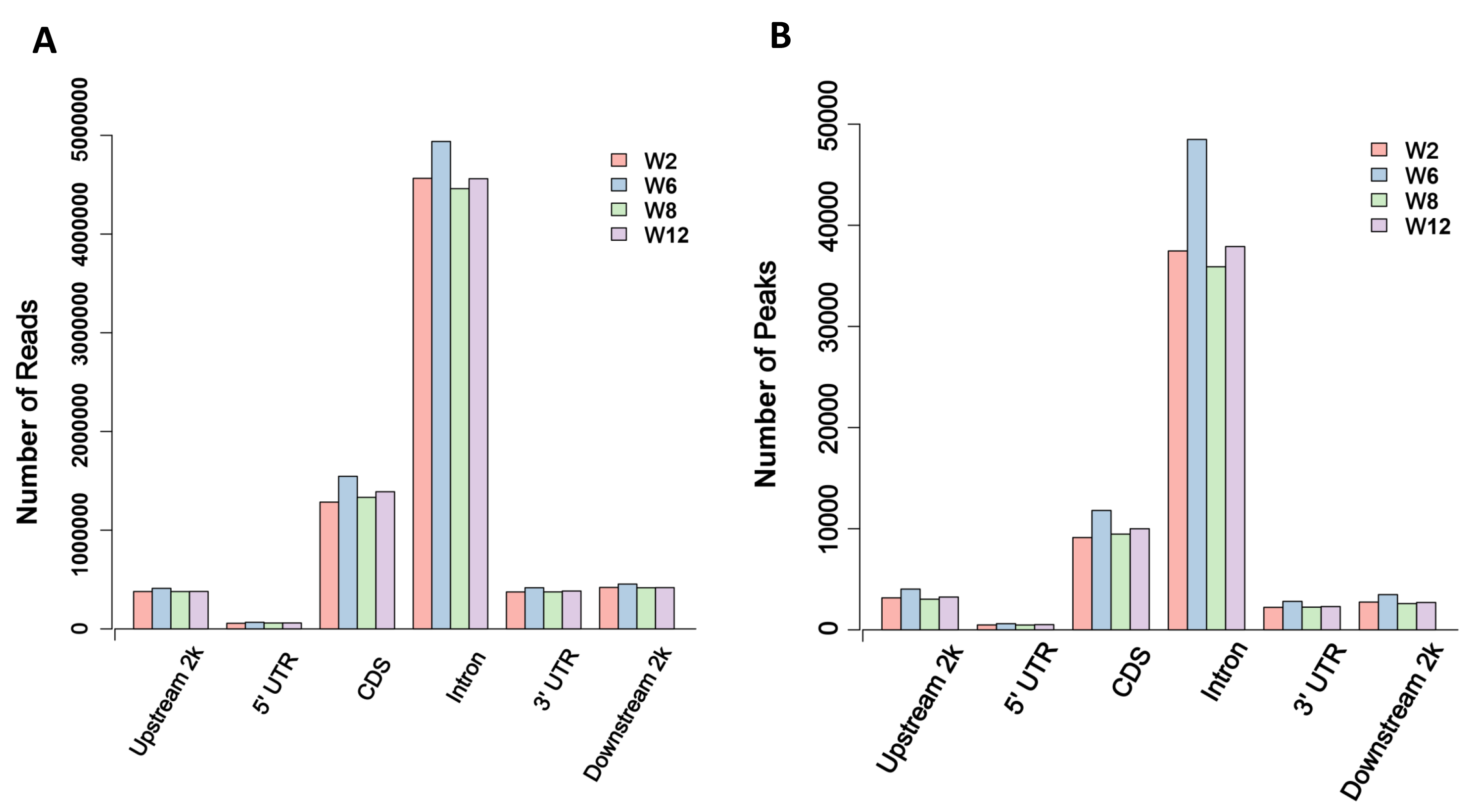

Supplement: Supplementary file 10 [file Image_5.TIF]

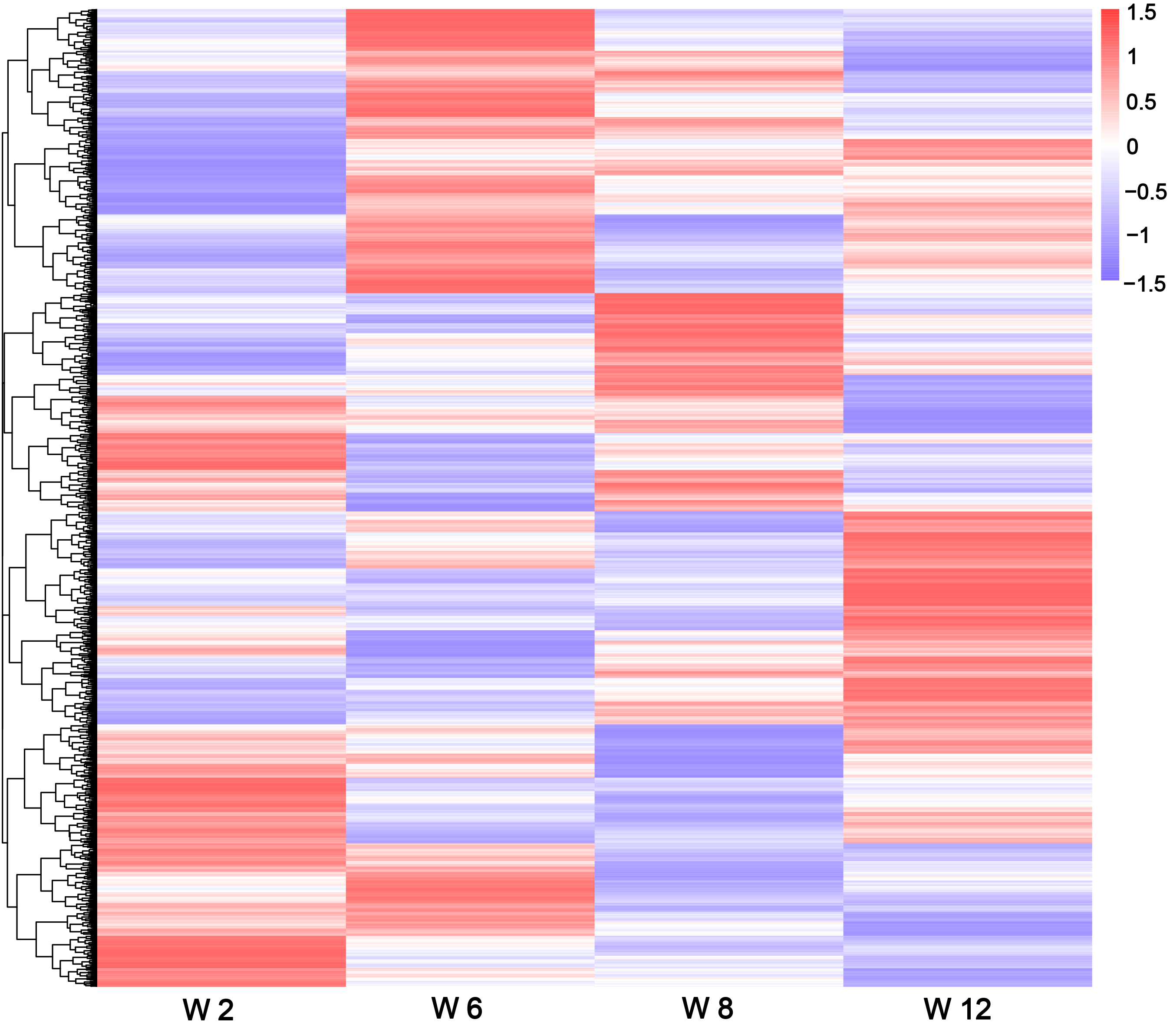

Supplement: Supplementary file 11 [file Image_6.TIF]
